# VirPLM: antigenic prediction of influenza A/H3N2 viruses with a fine-tuned protein language model

**DOI:** 10.1093/bioinformatics/btag692

**Published:** 2026-09-18

**Authors:** Xingyi Li, Kexin Xiao, Chunyan Zhou, Xiangting Jia, Dongmin Zhao, Jialuo Xu, Xianying Zeng, Jianzhong Shi, Xuequn Shang, Junnan Zhu, Huihui Kong

**Affiliations:** School of Computer Science, Northwestern Polytechnical University, Xi'an 710129, China; School of Computer Science, Northwestern Polytechnical University, Xi'an 710129, China; School of Computer Science, Northwestern Polytechnical University, Xi'an 710129, China; School of Computer Science, Northwestern Polytechnical University, Xi'an 710129, China; School of Computer Science, Northwestern Polytechnical University, Xi'an 710129, China; School of Computer Science, Northwestern Polytechnical University, Xi'an 710129, China; State Key Laboratory of Animal Disease Control and Prevention, Harbin Veterinary Research Institute, Chinese Academy of Agricultural Sciences, Harbin 150001, China; State Key Laboratory of Animal Disease Control and Prevention, Harbin Veterinary Research Institute, Chinese Academy of Agricultural Sciences, Harbin 150001, China; School of Computer Science, Northwestern Polytechnical University, Xi'an 710129, China; State Key Laboratory of Multimodal Artificial Intelligence Systems, Institute of Automation, Chinese Academy of Sciences, Beijing 100190, China; State Key Laboratory of Animal Disease Control and Prevention, Harbin Veterinary Research Institute, Chinese Academy of Agricultural Sciences, Harbin 150001, China

## Abstract

**Motivation:**

Human influenza A/H3N2 viruses undergo rapid antigenic evolution primarily driven by the hemagglutinin subunit 1 (HA1). Within HA1, amino acid substitutions under immune pressure cause antigenic drift, necessitating frequent updates to vaccine strains. While hemagglutination inhibition (HI) assays remain the gold standard for assessing antigenic relationships, their labor-intensive and low-throughput nature limits scalability. Fortunately, the rapid accumulation of HA1 sequences enables sequence-based antigenic prediction, yet effectively extracting informative representations from these viral sequences remains challenging.

**Results:**

In this study, we present VirPLM, a two-stage framework that adapts the ESM-2 protein language model to H3N2 HA1 sequences for antigenic prediction. VirPLM significantly outperforms representative methods and maintains robust performance under both cross-validation and retrospective time-split evaluations. Moreover, VirPLM identifies highly critical sites enriched in known regions related to antigenic evolution. In the season-specific coverage analysis, VirPLM-prioritized strains achieve higher estimated coverage rates than the corresponding historical strains recommended by the World Health Organization in most evaluated seasons, suggesting that VirPLM can provide complementary sequence-based evidence for candidate strain prioritization.

**Availability and Implementation:**

The source code is available at https://github.com/xingyili/VirPLM, and the version used in this study is archived in Zenodo (DOI: 10.5281/zenodo.21650323).

## 1 Introduction

Human influenza A/H3N2 virus is one of the major human influenza viruses and remains a persistent challenge to global public health due to its rapid antigenic evolution. The antigenicity of influenza A/H3N2 viruses is determined primarily by the hemagglutinin subunit 1 (HA1), which is a major target of neutralizing antibodies. Under host immune pressure, HA1 continuously accumulates amino acid substitutions that drive antigenic drift, reduce the protection conferred by pre-existing population immunity, and necessitate frequent vaccine strain updates (Wiley *et al.* 1981, Bouvier and Palese 2008, Koel *et al.* 2013, Petrova and Russell 2018, World Health Organization n.d).

Vaccination is the cornerstone of human influenza prevention and control and remains one of the most effective and cost-effective public health interventions against human influenza (Virelizier 1975, Sugishita and Sugawara 2021, Choi *et al*. 2022). However, vaccine effectiveness varies substantially across seasons, largely depending on the antigenic match between vaccine strains and the viruses that subsequently circulate (Gupta *et al.* 2006, Cai *et al.* 2012, Du *et al.* 2012, Petrova and Russell 2018, Rolfes *et al.* 2019, Chung *et al.* 2020, Tenforde *et al.* 2021). To guide vaccine strain selection, World Health Organization (WHO) vaccine composition meetings integrate global surveillance data to assess ongoing viral evolution and antigenic drift (Shah *et al*. 2024, World Health Organization n.d). Nevertheless, istorical vaccine mismatches underscore the inherent difficulties in formulating effective vaccine recommendations, even with such rigorous monitoring. Overcoming this rapid evolution to facilitate precise vaccine updates urgently requires proactive antigenic prediction.

Experimentally, antigenic prediction of H3N2 viruses still relies largely on the hemagglutination inhibition (HI) assay, which remains a gold standard for assessing viral antigenicity. However, it is labor-intensive, relatively low-throughput, and often unable to provide timely prediction of newly emerging strains (Cai *et al.* 2012, Pedersen 2014, Shah *et al.* 2024). The advent of high-throughput sequencing has driven a rapid accumulation of H3N2 HA1 sequence data, presenting a valuable opportunity for sequence-based antigenic prediction. Consequently, computational approaches have emerged to serve as robust alternatives to traditional HI-based assessments.

Several machine-learning frameworks have been developed to evaluate the antigenic relationships of virus pairs based on HA1 sequence differences. For instance, Shah *et al*. (2024) utilize an AAindex2 mutation matrix to encode amino acid substitutions, subsequently training an AdaBoost model for antigenic prediction. MFPAD (Li *et al.* 2024) employs binary encoding to represent sequence differences and integrates 12 types of antigenicity-related features to train an XGBoost model for predicting antigenic distance. To capture more complex patterns, other methods leverage deep-learning architectures to learn representations directly from HA1 sequences. For example, CNN-PSO (Lee *et al.* 2020) extracts paired features from selected HA sites using AAindex2 substitution and AAindex3 contact-potential matrices, feeding them into a particle swarm optimization (PSO)-optimized convolutional neural network (CNN) to classify antigenic variants. Furthermore, PREDAC-CNN (Meng *et al.* 2024) formulates paired HA1 sequences into an input matrix that combines amino acid identities with physicochemical properties to predict antigenic relationships. More recent studies explore attention-based and graph-based architectures for antigenic prediction. For example, FluAttn (Geng *et al.* 2026) and PREDAC-Transformer (Liu *et al.* 2025a) employ attention mechanisms to model dependencies among HA sequence sites, whereas FluGC (Liu *et al.* 2025b) formulates antigenic relationships among virus strains as a graph and combines graph contrastive learning with a graph attention network for antigenic prediction.

Despite these advancements, existing methods still exhibit limited capability in learning informative sequence representations, which intrinsically restricts their performance in antigenic prediction. Unlike models trained exclusively on influenza A HA1 sequences, protein language models (PLMs) such as ESM-2 are pretrained on large-scale protein sequence corpora through self-supervised learning. This paradigm enables them to capture complex contextual and evolutionary patterns, thereby extracting highly informative HA1 representations that better characterize the antigenic relationships between virus pairs.

Here, we present VirPLM, a two-stage framework for influenza A/H3N2 antigenic prediction based on an ESM-2 protein language model adapted to HA1 sequences (Fig. 1). In the first stage, ESM-2 is further trained on H3N2 HA1 sequences using a masked language modeling objective to adapt its representations to HA1 sequences. In the second stage, pairs of HA1 sequences are jointly encoded by the HA1-adapted ESM-2 encoder to learn pair-level representations for antigenic prediction. VirPLM achieves superior performance in both antigenic distance prediction and antigenic variant discrimination, exhibiting remarkable robustness under limited-data and retrospective time-split evaluation. Furthermore, the model identifies high-importance predictive sites that map precisely to known antigenic regions. Most notably, vaccine strains prioritized by VirPLM achieve higher coverage rates than historical WHO-recommended strains across most evaluated seasons.

## 2 Materials and methods

### 2.1 Dataset collection and preprocessing

#### 2.1.1 Data acquisition and curation

HA1 sequences of influenza A/H3N2 viruses collected from 1968 through April 2025 are obtained from the GISAID database (Shu and McCauley 2017) at https://gisaid.org/ and from the datasets curated by Smith *et al.* (2004). HI titer data are assembled from annual and interim reports issued by the Worldwide Influenza Centre at the Francis Crick Institute from 2003 through February 2025 (The Francis Crick Institute 2025) (https://www.crick.ac.uk/research/platforms-and-facilities/worldwide-influenza-centre/annual-and-interim-reports), together with the dataset curated by Smith *et al.* (2004), resulting in an initial collection of 126 249 HI titer values.

#### 2.1.2 Sequence alignment and quality control

Multiple sequence alignment (MSA) is performed using MAFFT, followed by quality control of the aligned sequences. Sequences containing ambiguous amino acid codes (B, Z, J, or X) or with a gap rate greater than 10% are removed. To reduce potential effects of sequencing artifacts, sequences with incomplete N- or C-termini are also excluded. The preprocessing procedure follows the default settings of the FluNexus platform (Li *et al.* 2026) (https://flunexus.com). After sequence alignment and quality control, 165 640 HA1 sequence records collected from 1968 through April 2025 are retained. This dataset includes 16 264 additional records collected from January through April 2025 for the postseason evaluation of the 2024NH season. To construct the HI-based antigenic prediction dataset, virus identity depends jointly on the strain name (including the location, isolate identifier, and collection year) and the passage information. Records sharing the same strain name but carrying different passage information are treated as distinct virus isolates. Records sharing both the same strain name and the same passage information are considered duplicates and deduplicated before sequence–HI matching, resulting in 6 316 HA1 sequence records with corresponding HI measurements. Sequence records without matched HI data are retained for virus-specific pretraining and the vaccine strain recommendation analysis.

#### 2.1.3 HI titer preprocessing

Before calculating antigenic distances, all HI records are processed using a common procedure independent of the antigenic-distance definition. Left-censored titers (e.g. “<40”) are excluded, whereas right-censored titers (e.g. “>512”) are doubled to approximate endpoint titers (Smith *et al.* 1999, Lee and Chen 2004, Smith *et al.* 2004, Meng *et al.* 2024). After this common preprocessing, the normalized antigenic distance (NAD) and the Archetti–Horsfall distance (AHD) are calculated separately according to their respective data requirements. When multiple independent antigenic-distance estimates are obtained for the same virus pair under a given distance definition, these estimates are averaged to obtain a single pair-level value.

#### 2.1.4 Quantification of antigenic distance

To quantify antigenic relationships, we employ both NAD (Lee and Chen 2004, Smith *et al.* 2004, Meng *et al.* 2024) and AHD (Cai *et al.* 2012, Liu *et al.* 2025b). The NAD between antigen *i* and antiserum *j* is defined as:


(1) $$D_{i,j}=b_{j}-{\;\text{log}\;}_{2}\left(H_{i,j}\right),\;b_{j}={\;\text{log}\;}_{2}\left(H_{j,j}\right),$$

where $H_{i,j}$ denotes the heterologous HI titer of antigen *i* against antiserum *j*, and $H_{j,j}$ denotes the experimentally measured homologous HI titer of reference antigen *j* against its corresponding antiserum *j*. This normalization accounts for differences in homologous titers across serum panels. Following previous studies, an NAD of at least 2 units, corresponding to a four-fold or greater reduction in HI titer, is used to define antigenic variants.

For viruses *i* and *j*, the AHD is defined as:


(2) $$D_{i,j}=\sqrt{\frac{H_{i,i}H_{j,j}}{H_{i,j}H_{j,i}}}$$

where $H_{i,i}$ and $H_{j,j}$ denote the homologous HI titers of viruses *i* and *j*, respectively, and $H_{i,j}$ and $H_{j,i}$ denote the reciprocal heterologous HI titers. Accordingly, AHD can be calculated only for virus pairs with a complete reciprocal HI titer set, comprising valid homologous titers for both viruses and the corresponding reciprocal heterologous titers. This requirement further implies that antisera must have been raised against both viruses in the pair. Following previous studies (Meng *et al.* 2024, Liu *et al.* 2025b), virus pairs with an AHD of at least 4 are defined as antigenic variants, whereas those with an AHD below 4 are considered antigenically similar.

### 2.2 Virus-specific pretraining for HA1 sequence representation learning

HA1 sequences of influenza A/H3N2 viruses evolve under constraints related to structural stability, receptor binding, and host immune pressure, leading to complex sequence variation. To adapt the protein language model ESM-2 (Lin *et al.* 2023) to H3N2 HA1 sequences, we further train the model on the curated HA1 sequence set.

Pretraining employs a masked language modeling (MLM) objective. In this task, a subset of amino acid sites is randomly masked, and the model is trained to recover the original amino acids from the remaining sequence context. Formally, given an amino acid sequence $X=(x_{1},\ldots ,x_{L})$ and a set of masked sites *M*, the model predicts the original amino acids at the masked sites. Let $p_{\theta }(x_{i}=a|X_{\setminus M})$ denote the probability assigned by the model to amino acid *a* at site *i*. The MLM loss is the average negative log-likelihood over masked sites:


(3) $$L_{\text{MLM}}(\theta )=-\frac{1}{\left|M\right|}\sum_{i\in M}\;\text{log}\;p_{\theta }(x_{i}|X_{\setminus M}).$$

### 2.3 Joint representation learning of paired virus sequences using a cross-encoder

To model antigenic relationships between virus pairs, we use a cross-encoder architecture in which two HA1 sequences are concatenated and provided as a single input to the HA1-adapted ESM-2 encoder. This design allows the encoder to process the two sequences jointly rather than independently.

After joint encoding, the [CLS] representation from the final hidden layer is used as a pair-level representation, followed by a single-layer linear regressor. The regressor is trained with mean squared error (MSE) loss to predict antigenic distances derived from HI assays. Specifically, given two HA1 sequences $S^{\left(a\right)}$ and $S^{\left(b\right)}$, we concatenate them as $S^{\left(a\right)}[\mathrm{SEP}]S^{\left(b\right)}$ and feed the resulting paired input into the HA1-adapted ESM-2 encoder. From the final hidden layer, we extract the [CLS] token representation as a global representation vector $h_{\text{cls}}\in R^{d}$, where *d* denotes the transformer’s hidden size. The single-layer linear regressor maps $h_{\text{cls}}$ to a scalar prediction $\hat{y}$, corresponding to the predicted antigenic distance:


(4) $$\hat{y}=w^{⊤}h_{\text{cls}}+b,\;w\in R^{d},\;b\in R.$$

Given a training set of *N* paired samples ${\left\{(S_{i}^{\left(a\right)},S_{i}^{\left(b\right)},y_{i})\right\}}_{i=1}^{N}$, we train VirPLM by minimizing the MSE loss:


(5) $$L_{\text{MSE}}(\theta ,w,b)=\frac{1}{N}\sum_{i=1}^{N}{\left(y_{i}-{\hat{y}}_{i}\right)}^{2},$$

where θ denotes the transformer parameters and (w,b) are the regressor parameters. For antigenic variant discrimination, a threshold *t* is applied to the predicted antigenic distance $\hat{y}$. We use t=2 for NAD and t=4 for AHD, consistent with the corresponding definitions of antigenic variants. The predicted and observed binary labels, denoted by $\hat{c}$ and *c*, respectively, are defined as:


(6) $$\hat{c}=1\{\hat{y}\geq t\},\;c=1\{y\geq t\},$$

where 1{·} denotes the indicator function. Accordingly, virus pairs with an NAD of at least 2 or an AHD of at least 4 are defined as antigenic variants, whereas those below the corresponding threshold are defined as antigenically similar.

### 2.4 Site importance scores

To estimate the contribution of individual HA1 sites to antigenic prediction, we perform a site-wise masking analysis. For each antigen–antiserum pair *j*, let $x^{\left(j\right)}$ denote the HA1 sequence of the antigen virus and $y^{\left(j\right)}$ denote the HA1 sequence of the reference virus corresponding to the antiserum. The unmasked antigen–antiserum pair is represented as $\left(x^{\left(j\right)},y^{\left(j\right)}\right)$, and $s(x^{\left(j\right)},y^{\left(j\right)})$ denotes the scalar prediction produced by VirPLM trained on NAD-derived antigenic distances. We then mask site *i* separately in the two HA1 sequences, yielding $\left(x_{\text{mask}(i)}^{\left(j\right)},y^{\left(j\right)}\right)$ for antigen masking and $\left(x^{\left(j\right)},y_{\text{mask}(i)}^{\left(j\right)}\right)$ for antiserum masking. The importance of site *i* is defined as the average absolute change in the scalar prediction score after the two single-site masking operations:


(7) $$s_{0}^{\left(j\right)}=s\left(x^{\left(j\right)},y^{\left(j\right)}\right),$$

(8) $$\begin{array}{cc} I_{i}^{\left(j\right)}=\frac{1}{2}( & \left|s\left(x_{\text{mask}(i)}^{\left(j\right)},y^{\left(j\right)}\right)-s_{0}^{\left(j\right)}\right|\\ & +\left|s\left(x^{\left(j\right)},y_{\text{mask}(i)}^{\left(j\right)}\right)-s_{0}^{\left(j\right)}\right|). \end{array}$$

Population-level importance scores for each site are obtained by averaging $I_{i}^{\left(j\right)}$ over all samples:


(9) $$I_{i}=\frac{1}{N}\sum_{j=1}^{N}I_{i}^{\left(j\right)}.$$

The resulting vector *I* is used to rank HA1 sites according to their effects on model predictions. A higher $I_{i}$ value indicates a larger change in the prediction when site *i* is masked. We focus subsequent analyses on the top 30 ranked sites.

### 2.5 Regional enrichment analysis of top-ranked sites

To examine whether the high-ranked sites identified by VirPLM are over-represented in known antigenicity-related regions of HA1, we perform a regional enrichment analysis using the top 30 sites. The tested regions include the five major antigenic epitope regions A–E. For each known region, enrichment is evaluated using a one-sided Fisher’s exact test, and the corresponding *P*-value is defined as:


(10) $$P=\sum_{x=a}^{\text{min}(K,M)}\frac{\left(\begin{array}{c} M\\ x \end{array}\right)\left(\begin{array}{c} N-M\\ K-x \end{array}\right)}{\left(\begin{array}{c} N\\ K \end{array}\right)}.$$

Here, *N* denotes the total length of the HA1 sequence (328), *K* denotes the number of top-ranked sites selected (30 in this study), *M* denotes the number of sites in the known region, and *a* denotes the number of shared sites between the top-*K* sites and the known region. Under the null hypothesis that top-*K* is obtained by randomly selecting *K* sites from the *N* candidates, the *P*-value is the probability of observing at least *a* sites within the known region. Regions with *P* < .05 are considered significantly enriched among the top 30 sites.

### 2.6 Definition of coverage rate

Vaccine effectiveness depends in part on the antigenic match between the vaccine strain and circulating viruses in the target season. Because viruses circulating in the target season are not fully observed at the time of strain recommendation, candidate selection can only use surveillance data available before the corresponding recommendation deadline. Under this setting, we use the coverage rate to quantify the proportion of circulating viruses in a given evaluation set that are antigenically close to a candidate vaccine strain.

In this study, the coverage threshold τ is set to 2 antigenic units, consistent with the threshold used for antigenic variant discrimination. Since antigenic distance is defined on a ${\;\text{log}\;}_{2}$-transformed HI titer scale, a distance of 2 corresponds to a four-fold reduction in HI titer, a widely accepted indicator of significant antigenic difference (Smith *et al.* 1999, Lee and Chen 2004, Smith *et al.* 2004, Meng *et al.* 2024). Accordingly, a candidate vaccine strain is considered to cover a target virus provided their predicted antigenic distance strictly falls below 2. Prior to coverage estimation, records with identical HA1 sequences are merged, with their observation counts preserved as frequency weights. When an identical candidate HA1 sequence is associated with multiple strain names, the first recorded designation is retained.

Let *S* denote the set of circulating viruses in a given evaluation period. For each s∈S, let $w_{s}$ denote its frequency weight, and let $d_{M}(v,s)$ be the NAD between candidate strain *v* and circulating virus *s*. The coverage rate is defined as:


(11) $$\text{coverage}\;\text{rate}(v,S)=\frac{\sum_{s\in S}w_{s}I\left(d_{M}(v,s)<\tau \right)}{\sum_{s\in S}w_{s}}.$$

where I(·) is the indicator function. A higher coverage rate indicates that a larger proportion of circulating viruses in the target season are predicted to be antigenically similar to the candidate strain under the four-fold HI titer criterion.

The frequency-weighted mean antigenic distance is calculated as:


(12) $${\bar{d}}_{M}(v,S)=\frac{\sum_{s\in S}w_{s}d_{M}(v,s)}{\sum_{s\in S}w_{s}}.$$

Candidate strains are ranked primarily in descending order of their coverage rates. In cases of equivalent coverage, candidates exhibiting a lower frequency-weighted mean antigenic distance are prioritized.

During candidate prioritization, antigenic distances are predicted using VirPLM, which is trained exclusively on data available before the corresponding fixed decision-time cutoff. In the subsequent postseason evaluation, experimentally measured NAD values are utilized whenever available, whereas the corresponding postseason model is used exclusively to estimate missing experimental distances. The aforementioned coverage metric is then uniformly applied to benchmark the VirPLM-prioritized strain against the historical WHO-recommended vaccine strain.

### 2.7 Implementation and evaluation

VirPLM is implemented in PyTorch. Its encoder is initialized with ESM-2 (Lin *et al.* 2023), a protein language model with approximately 150 million parameters. The model is trained in two stages. First, the encoder is further trained on HA1 sequences using the MLM objective to adapt its representations to H3N2 HA1 sequences. The model is optimized using the AdamW optimizer for 10 epochs, with a learning rate of $5\times 10^{-4}$, a batch size of 16, a weight decay of 0.01, and four gradient accumulation steps. The MLM masking probability is set to 0.15, a linear learning-rate schedule with a warm-up ratio of 0.1 is applied, and gradients are clipped to a maximum norm of 1.0. In the second stage, the pretrained encoder is retained and fine-tuned using inputs of paired HA1 sequences to predict antigenic distance. Specifically, a single-layer linear head is attached to produce a scalar prediction of antigenic distance. The fine-tuning process utilizes the AdamW optimizer for a maximum of 50 epochs, with a learning rate of $5\times 10^{-5}$ and a training batch size of 8. To promote stable convergence, the learning rate is halved if the validation root mean square error (RMSE) fails to improve for three consecutive epochs, while early stopping is applied with a patience of 10 epochs. The random seed is set to 3407 for both training stages.

To comprehensively evaluate model performance, we implement two complementary validation strategies. A five-fold cross-validation is used to assess predictive performance under random data splits, whereas a retrospective time-split evaluation is conducted on a seasonal basis, relying exclusively on historical data available prior to each target season to simulate real-world scenarios. The retrospective time-split evaluation includes 26 test seasons from 2012NH to 2024SH. For each test season, virus-specific MLM pretraining and supervised fine-tuning are performed independently using only HA1 sequence records and HI data satisfying the corresponding temporal cutoff. Detailed temporal partitioning rules for the retrospective time-split evaluation are provided in Supplementary Note S2.1.

For both evaluation strategies, the overall performance is calculated as a sample-weighted average across evaluation units:


(13) $$M_{\text{avg}}=\frac{1}{\sum_{k\in K}n_{k}}\sum_{k\in K}n_{k}M_{k},$$

where *k* denotes an evaluation unit, corresponding to a test fold in five-fold cross-validation or a test season in retrospective evaluation. *K* denotes the set of all evaluation units, $n_{k}$ denotes the number of test samples in unit *k*, and $M_{k}$ denotes the metric value computed for that unit.

Let $D_{ij}$ denote the observed antigenic distance between antigen *i* and antiserum *j* derived from HI titers, and let ${\hat{D}}_{ij}$ denote the corresponding predicted value. If evaluation unit *k* contains $n_{k}$ antigen–antiserum pairs, MSE for that unit is defined as:


(14) $${\text{MSE}}_{k}=\frac{1}{n_{k}}\sum {\left(D_{ij}-{\hat{D}}_{ij}\right)}^{2}.$$

The corresponding RMSE and mean absolute error (MAE) values are defined as:


(15) $$\begin{array}{c} {\text{RMSE}}_{k}=\sqrt{{\text{MSE}}_{k}},\\ {\text{MAE}}_{k}=\frac{1}{n_{k}}\sum |D_{ij}-{\hat{D}}_{ij}|. \end{array}$$

For fold *k* or test season *k*, let ${\text{TP}}_{k},{\text{FP}}_{k},{\text{FN}}_{k},{\text{TN}}_{k}$ denote the confusion-matrix counts. Precision is defined as:


(16) $${\text{Precision}}_{k}=\frac{{\text{TP}}_{k}}{{\text{TP}}_{k}+{\text{FP}}_{k}},$$

Recall and the false-positive rate (FPR) are defined as:


(17) $${\text{Recall}}_{k}=\frac{{\text{TP}}_{k}}{{\text{TP}}_{k}+{\text{FN}}_{k}},$$

(18) $${\text{FPR}}_{k}=\frac{{\text{FP}}_{k}}{{\text{FP}}_{k}+{\text{TN}}_{k}}.$$

The area under the receiver operating characteristic curve (AUC) and the area under the precision–recall curve (AUPRC) are defined as follows, where *R* denotes recall:


(19) $${\text{AUC}}_{k}=\int_{0}^{1}{\text{Recall}}_{k}(\text{FPR})\text{dFPR},$$

(20) $${\text{AUPRC}}_{k}=\int_{0}^{1}{\text{Precision}}_{k}(R)dR,$$

### 2.8 Benchmarked models

We evaluate the performance of VirPLM by comparing it with two baseline models and eight representative methods. Specifically, the baseline models include support vector machine (SVM) and multilayer perceptron (MLP), while conventional machine learning approaches are represented by AdaBoost (Shah *et al.* 2024), MFPAD (Li *et al.* 2024), and PREDAC (Du *et al.* 2012). Furthermore, deep learning comparisons incorporate CNN-PSO (Lee *et al.* 2020), PREDAC-CNN (Meng *et al.* 2024), FluAttn (Geng *et al.* 2026), PREDAC-Transformer (Liu *et al.* 2025a) and FluGC (Liu *et al.* 2025b). To ensure unbiased benchmarking, all the models are implemented in strict accordance with their original architectures where feasible, or otherwise adapted to the same training setting for comparison.

Methods outputting continuous antigenic distances are evaluated using both regression metrics and threshold-derived classification metrics. In contrast, models specifically designed for antigenic variant classification—namely CNN-PSO, PREDAC, PREDAC-CNN, and PREDAC-Transformer—are evaluated solely on classification performance. Comprehensive details regarding feature construction, architectural designs, and hyperparameter configurations are provided in Supplementary Note S1.

## 3 Results

### 3.1 Cross-validation evaluation of VirPLM for H3N2 antigenic prediction

To systematically evaluate the antigenic predictive performance of VirPLM for influenza A/H3N2, we conduct a five-fold cross-validation against ten representative methods, including AdaBoost (Shah *et al.* 2024), CNN-PSO (Lee *et al.* 2020), MFPAD (Li *et al.* 2024), PREDAC-CNN (Meng *et al.* 2024), PREDAC (Du *et al.* 2012), FluAttn (Geng *et al.* 2026), FluGC (Liu *et al.* 2025b), PREDAC-Transformer (Liu *et al.* 2025a), MLP, and SVM. For the classification task, VirPLM demonstrates exceptional capability in discriminating antigenic variants. Specifically, when utilizing the NAD metric, VirPLM maintains robust performance despite the class imbalance of the dataset (Fig. S1; Fig. 2a, Precision = 0.925, AUC = 0.898, and AUPRC = 0.939). In the regression task, VirPLM minimizes prediction errors (Fig. 2b, MAE = 0.687, MSE = 0.799, RMSE = 0.894). This superiority extends to evaluations utilizing the AHD metric, where VirPLM exhibits superior discriminative power against all compared methods (Fig. S2, Precision = 0.822, AUC = 0.908, and AUPRC = 0.858) while concurrently generating highly accurate antigenic distance estimations (Fig. S2, MAE = 1.978, MSE = 15.144, and RMSE = 3.891).

**Figure 1:**
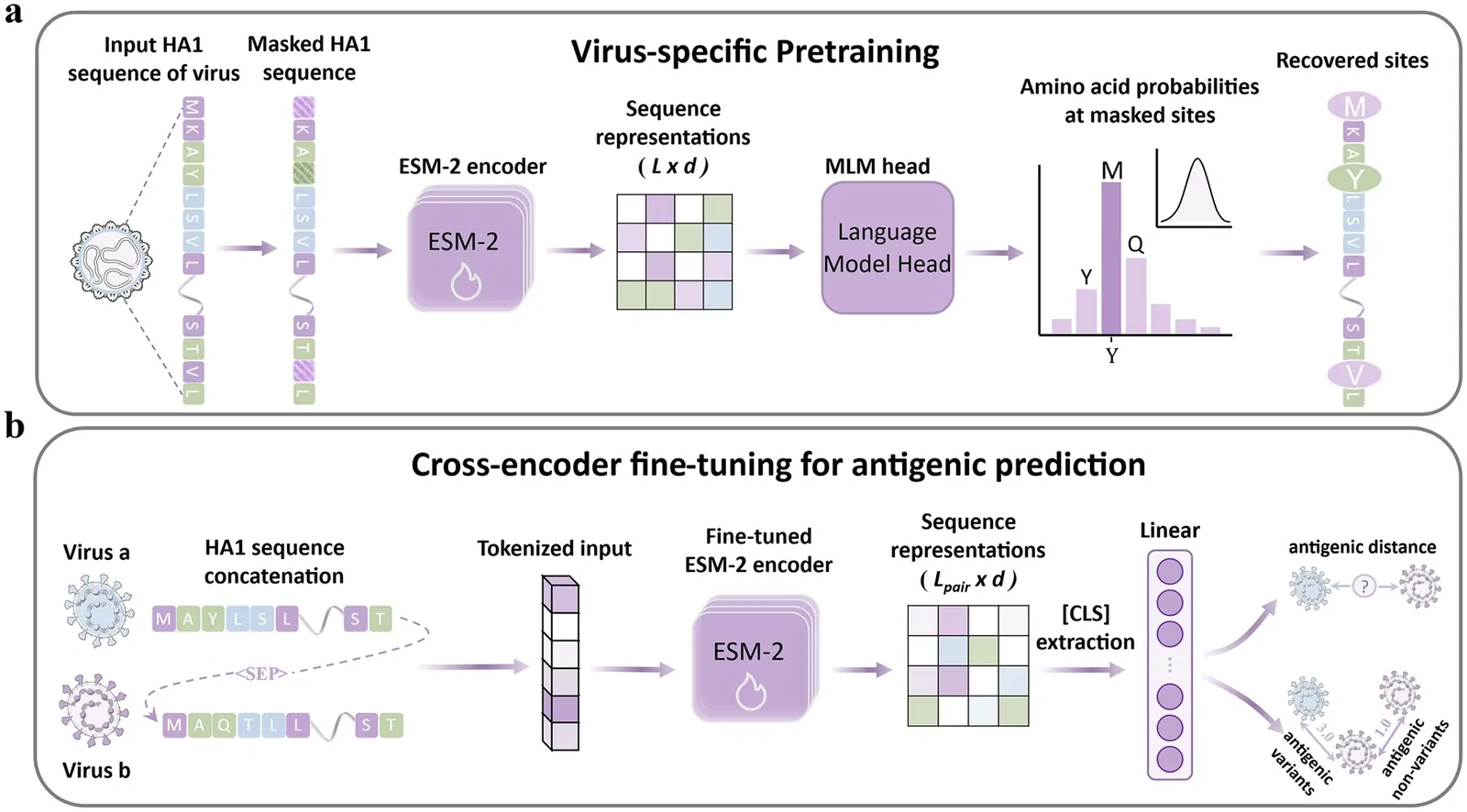
Overview of VirPLM. VirPLM is a two-stage framework for antigenic prediction based on HA1 sequence representation learning. (a) Virus-specific pretraining. ESM-2 is pretrained on influenza A/H3N2 HA1 sequences using MLM to learn HA1-specific contextual representations. (b) Antigenic prediction using a cross-encoder. Pairs of HA1 sequences are jointly encoded by the HA1-adapted ESM-2 encoder, and the resulting pair-level representation is fed into a regression head to predict antigenic distances measured by HI assays. The predicted antigenic distance can also be thresholded to discriminate antigenic variants from non-variants.

**Figure 2:**
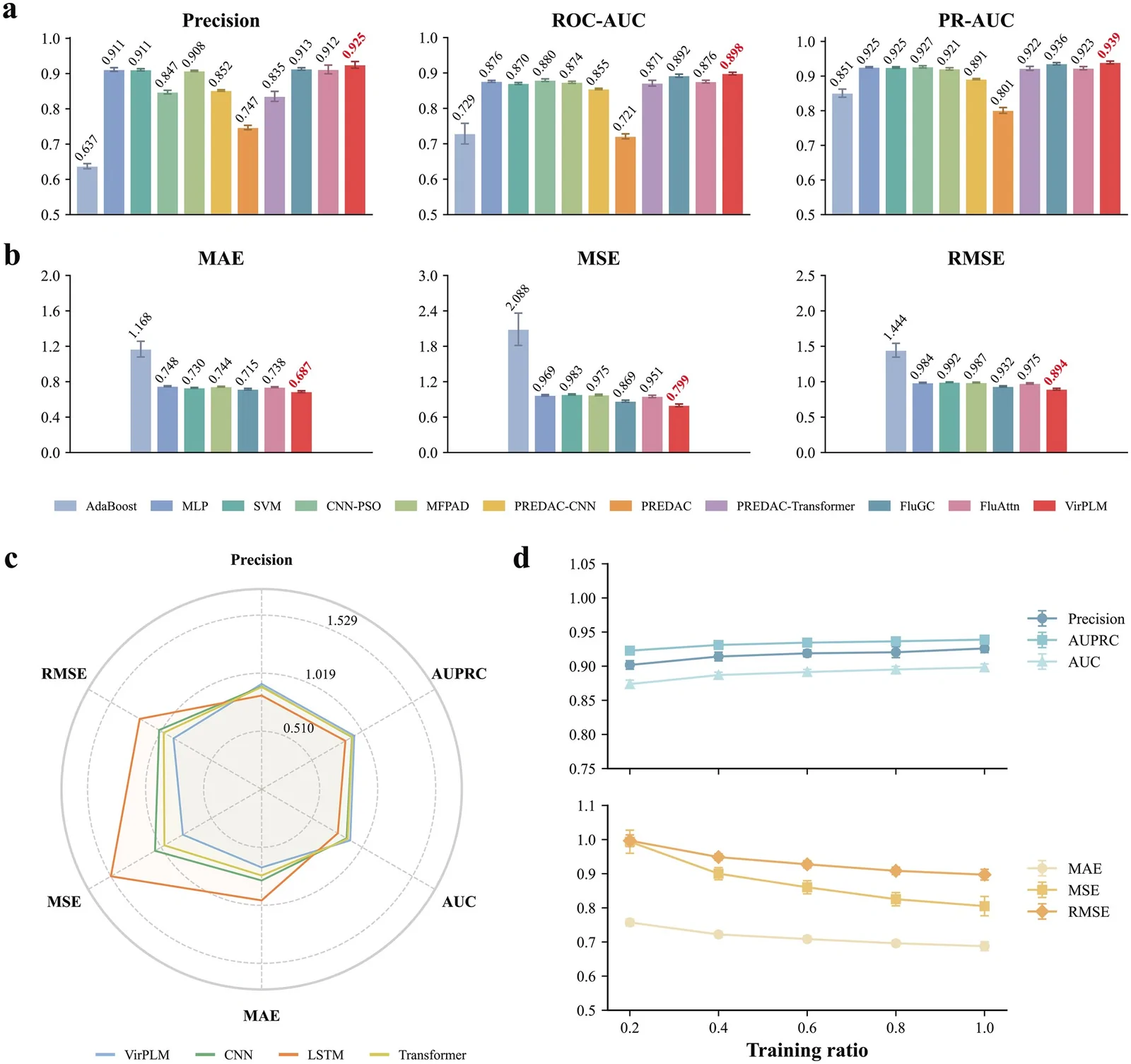
Performance evaluation of VirPLM for H3N2 antigenic prediction. (a) Comparative performance of VirPLM and ten benchmark methods (AdaBoost, MLP, SVM, CNN-PSO, MFPAD, PREDAC-CNN, PREDAC, FluAttn, FluGC, and PREDAC-Transformer) in discriminating antigenic variants from non-variants. (b) Comparative performance of VirPLM and six benchmark methods (AdaBoost, MLP, SVM, MFPAD, FluAttn, and FluGC) in predicting antigenic distance. (c) Ablation study of backbone encoders. (d) Robustness evaluation of VirPLM with a limited amount of training data.

These results indicate that the advantage of VirPLM arises from its virus-specific pretraining and pair-modeling strategy. Specifically, H3N2-specific MLM further adapts ESM-2 to the evolutionary patterns of HA1 sequences, while end-to-end supervised fine-tuning refines the encoder toward antigenicity-relevant signals. The pairwise cross-encoder subsequently contextualizes the two HA1 sequences jointly, enabling direct modeling of their inter-sequence dependencies and the sequence relationships underlying HI-derived antigenic distance. Together, these components yield task-specific pair representations that are more informative than fixed embeddings, manually constructed substitution features, or representations primarily derived from local sequence patterns.

To quantify the contribution of the ESM-2 backbone, we conduct an ablation study by substituting it with CNN, long short-term memory (LSTM), and Transformer architectures (Fig. 2c). The results show that the default VirPLM framework consistently outperforms these variants across all six evaluation metrics. These findings underscore that the pretrained protein language model backbone captures richer, more informative sequence representations, which are crucial for accurate H3N2 antigenic prediction.

We further assess VirPLM’s robustness under limited training data while keeping the test set fixed (Fig. 2d). As the training data is randomly subsampled from 100% down to 20%, the model’s classification performance exhibits remarkable stability, with negligible declines in AUC and AUPRC. While regression errors increase marginally, the overall predictive capacity remains highly robust.

### 3.2 Retrospective evaluation of VirPLM for H3N2 antigenic prediction

The timely identification of emerging antigenic variants and the precise characterization of circulating viral strains are critical for proactive pandemic surveillance and informed vaccine strain selection. To align our evaluation with these public health imperatives, we implement a retrospective testing strategy. Within this framework, models are trained separately for each of the 26 test seasons from 2012NH to 2024SH, strictly utilizing HA1 sequences and HI data accessible prior to the target season, ensuring an unbiased evaluation on the unseen seasonal data. This strategy mimics the real-world surveillance scenario, where the antigenic prediction of newly emerging strains depends entirely on historically available data. Given that AHD requires a complete reciprocal HI titer set, the resulting seasonal subsets are sparse and unevenly distributed (Fig. S4). Moreover, AHD is not a routine metric for antigenic distance in the WHO vaccine composition framework (Shah *et al*. 2024, World Health Organization 2024). Consequently, we conduct the retrospective time-split evaluation using NAD. The numbers of training and test pairs in each test season are shown in Fig. S3.

Under the retrospective time-split setting, VirPLM maintains consistently strong classification performance and the lowest regression errors among all benchmarked methods (Fig. 3). Such temporal robustness is vital for influenza surveillance and vaccine strain selection. As ongoing antigenic drift progressively distances circulating viruses from established vaccine strains, promptly detecting these divergences is crucial for informing vaccine update decisions (Suzuki 2015, Petrova and Russell 2018, Castro *et al.* 2020, Huddleston *et al.* 2020, Perofsky and Nelson 2020). The strong temporal predictive performance demonstrated by VirPLM suggests that it successfully captures intrinsic antigenic features, thereby highlighting its practical utility for tracking antigenic evolution in newly emerging viral variants.

**Figure 3:**
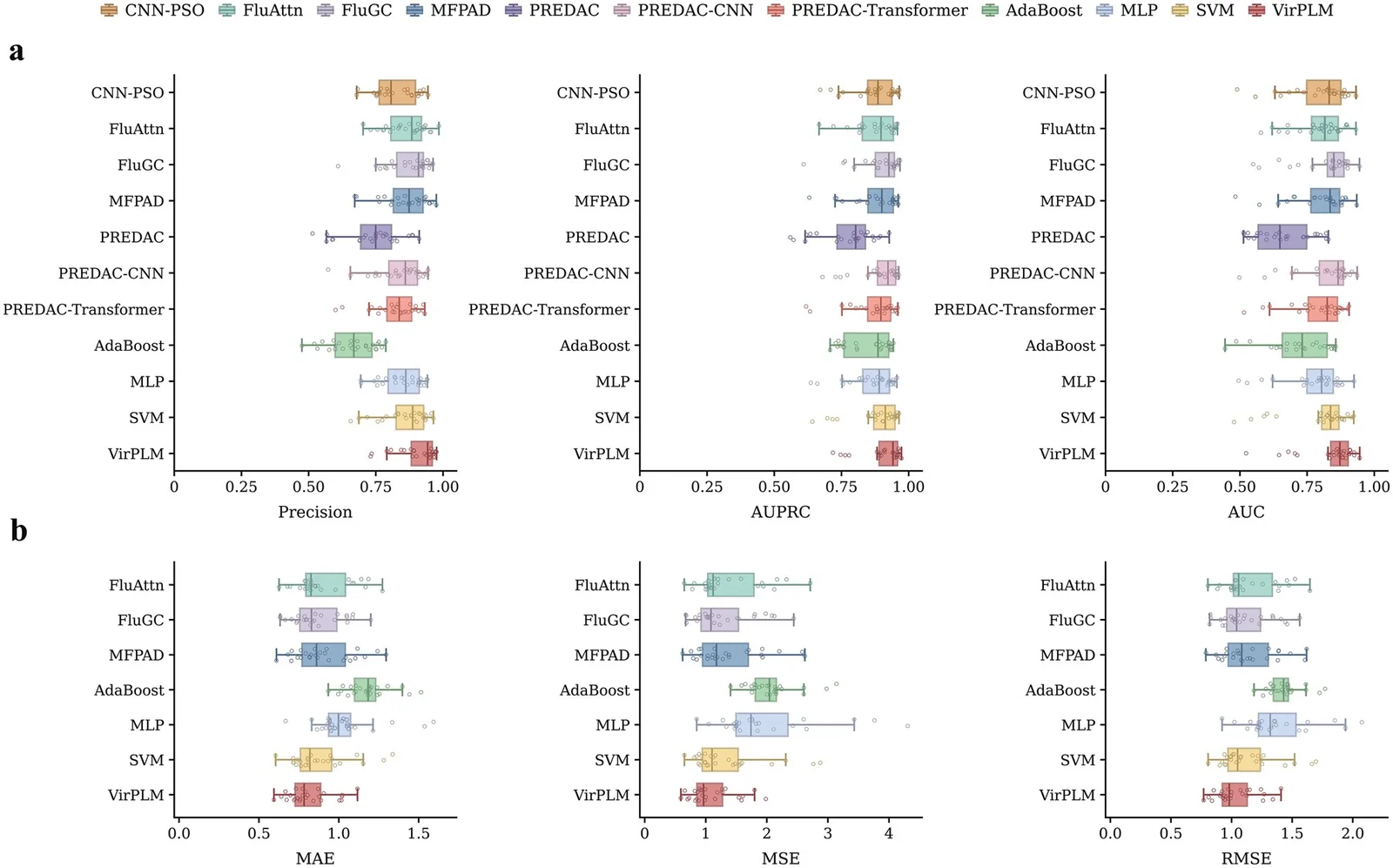
Retrospective evaluation of VirPLM for H3N2 antigenic prediction. (a) Classification performance in the retrospective setting, measured by Precision, AUPRC, and AUC across 26 test seasons (2012NH–2024SH). For each test season, training uses only data collected prior to that season, and evaluation is performed on samples from the target season. (b) Regression performance in the retrospective setting, measured by MAE, MSE, and RMSE. Individual data points in the boxplots indicate specific season outcomes.

### 3.3 VirPLM highlights biologically relevant HA1 sites associated with H3N2 antigenicity

Antigenic evolution in H3N2 influenza viruses is often driven by substitutions at a number of key sites in the HA1 sequences (Shih *et al.* 2007, Koel *et al.* 2013). To evaluate the biological interpretability of VirPLM, we perform a site-wise masking analysis across HA1 and examine the high-importance sites identified by VirPLM.

Previous antigenic studies have defined several HA1 regions closely associated with H3N2 antigenic variation, including the five major antigenic epitopes A–E on the HA1 globular head, the receptor-binding site (RBS) and its structural elements such as the 130-loop, 190-helix, and 220-loop, Koel’s sites, and glycosylation-related sites (Wiley *et al.* 1981, Popova *et al.* 2012, Koel *et al.* 2013, Jorquera *et al.* 2019). Notably, epitopes A and B are considered the primary determinants driving H3N2 antigenic evolution (Wiley *et al.* 1981, Popova *et al.* 2012, Wu *et al.* 2017). Furthermore, Koel *et al.* (2013) have demonstrated that a small number of substitutions can determine major antigenic cluster transitions in H3N2 viruses. Additionally, alterations in glycosylation sequons can reshape local antigenic surfaces and alter antibody accessibility, thereby contributing to H3N2 antigenic drift (Zost *et al.* 2017, Jorquera *et al.* 2019, Gouma *et al.* 2020).

The results show that the high-importance sites identified by VirPLM are concentrated in known regions associated with H3N2 antigenic variation (Fig. 4a). Consistently, enrichment analysis reveals that the top 30 sites prioritized by VirPLM are significantly enriched in Koel’s sites and epitopes A and B (Fig. 4b). Notably, several top-ranked sites identified by VirPLM have been previously associated with H3N2 antigenic evolution (Fig. 4c). For example, sites 145, 156, 158, 159, and 189 have been reported as key or accessory determinants of major antigenic cluster transitions in H3N2 viruses (Koel *et al.* 2013). Sites 158 and 160 are located in antigenic site B and are associated with glycosylation changes that can affect antibody recognition (Zost *et al.* 2017, Gouma *et al.* 2020). Site 186 is also located near the RBS and has been linked to receptor-binding properties and antigenic drift in H3N2 viruses (Lei *et al.* 2024). In addition, sites 128 and 135 are located in antigenic sites B and A, respectively, and have been associated with glycosylation remodeling and antigenic drift (Jorquera *et al.* 2019).

**Figure 4:**
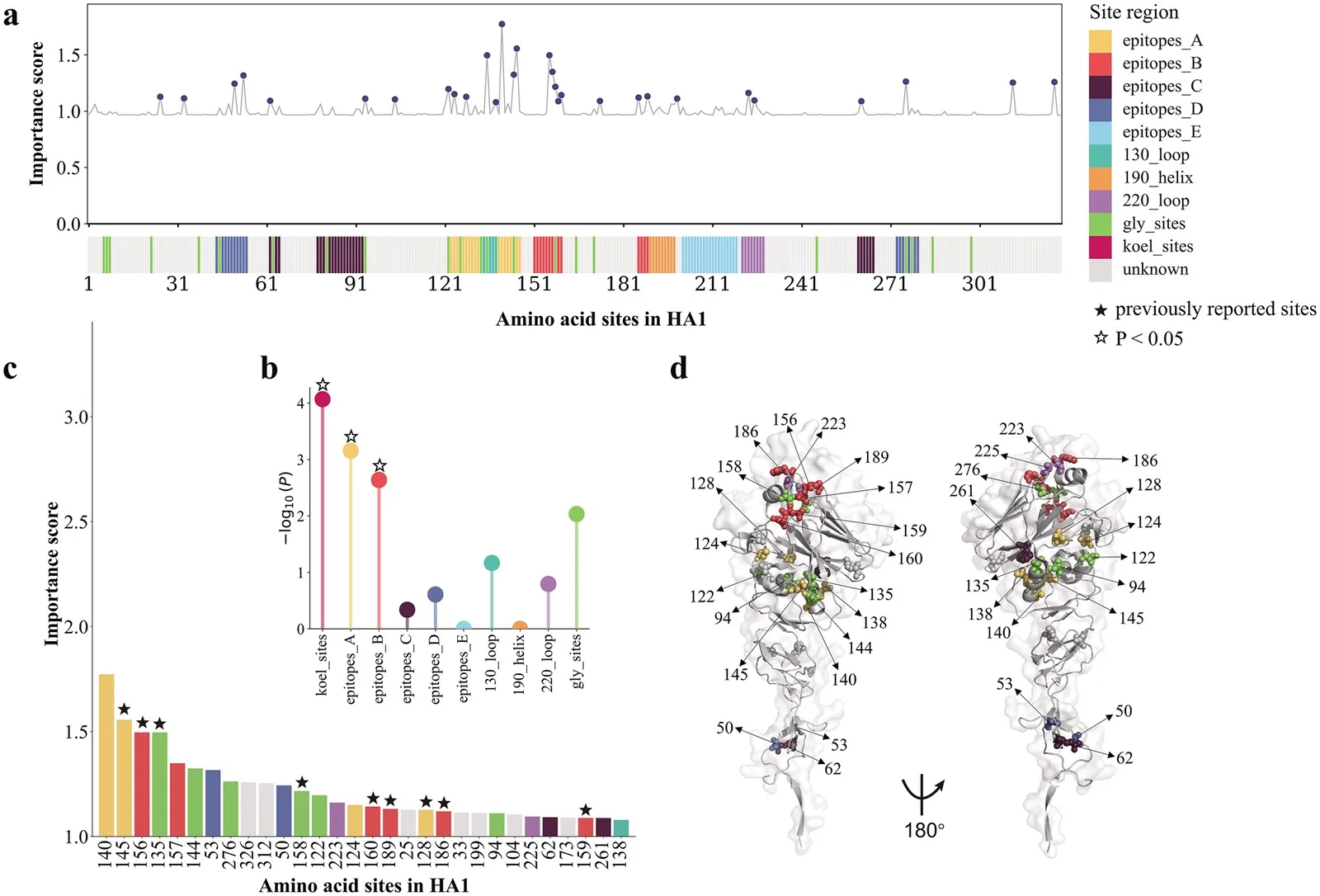
Site importance analysis of H3N2 HA1 using VirPLM. (a) Site-wise importance scores across HA1 amino acid sites derived from masking analysis. The top 30 sites with the highest importance scores are highlighted as blue dots. The annotation track below indicates the functional region corresponding to each site, and sites outside annotated functional regions are classified as ‘unknown’. (b) Enrichment analysis of the top 30 sites across known functional regions. Statistical significance is shown as $-{\;\text{log}\;}_{10}(P)$, and open stars indicate P<0.05. (c) Importance scores of the top 30 sites identified by VirPLM. Previously reported sites are marked with black stars. (d) Structural mapping of the top 30 sites onto the HA1 protein for H3N2 (Protein Data Bank accession: 4KVN; A/Perth/16/2009), shown in two views separated by a 180° rotation. Sites are color-coded according to their annotated functional regions.

Structural mapping shows that the top 30 sites are predominantly exposed on the HA1 globular head and clustered around the receptor-binding region (Fig. 4d). Together, sequence-level enrichment, literature-supported site annotations, and structural localization indicate that the sites prioritized by VirPLM are consistent with known biological determinants of H3N2 antigenic variation.

### 3.4 VirPLM supports vaccine strain prioritization based on coverage rates

The ultimate application of antigenic prediction is to support vaccine strain selection by pinpointing candidates with optimal antigenic matches to circulating viruses (Gupta *et al.* 2006, Cai *et al.* 2012, Suzuki 2015). Given that vaccine composition decisions inherently precede the target influenza season, we evaluate the capacity of VirPLM to inform this proactive selection by implementing a retrospective time-split framework. We set the data cutoffs to 1 February and 1 September, corresponding to the months in which the WHO vaccine composition consultations are generally held for the northern and southern hemispheres, respectively (World Health Organization 2023, 2024).

Candidate strain prioritization and postseason performance evaluation are executed as two strictly independent phases. For each target season, the decision-time model, the candidate strain pool, and the surveillance viruses utilized for candidate ranking are restricted by the corresponding cutoff date. To preclude temporal data leakage, viruses emerging in the subsequent target season are used exclusively for retrospective performance assessment. Comprehensive details regarding the periods used to construct the candidate pools, the model-training cutoffs, and the postseason evaluation windows are provided in Supplementary Notes S2.2 and S2.3. The strains prioritized by VirPLM and the corresponding historical WHO-recommended strains are listed in Table S1.

Across the evaluated seasons, the strains prioritized by VirPLM achieve higher coverage rates than the WHO-recommended strains in most seasons (Fig. 5). This improvement is particularly evident in 2014NH, when the WHO-recommended strain shows markedly reduced coverage, consistent with the well-documented vaccine mismatch and reduced vaccine effectiveness during the 2014–2015 H3N2 influenza season (Skowronski *et al.* 2016, Zimmerman *et al.* 2016). In contrast, the strain selected by VirPLM achieves substantially higher coverage in the same year, suggesting that VirPLM can provide complementary information for prioritizing candidate vaccine strains under rapid antigenic drift.

**Figure 5:**
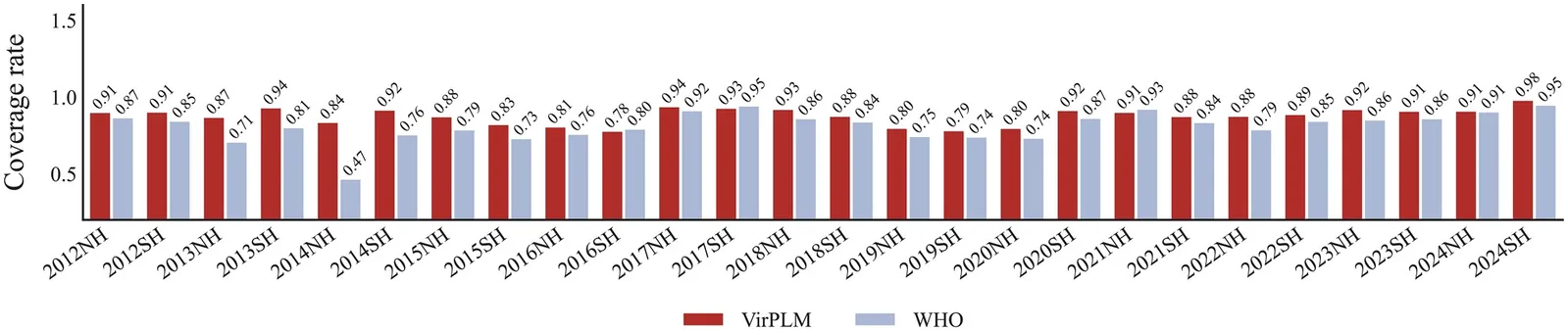
Coverage-rate evaluation of vaccine strain recommendations for H3N2. Vaccine coverage rates of strains prioritized by VirPLM and the corresponding historical WHO-recommended strains across target seasons under the retrospective recommendation framework.

## 4 Discussion

The rapid antigenic evolution of influenza A/H3N2 viruses remains a major challenge for global surveillance and vaccine strain selection. To address this, we develop VirPLM, a two-stage framework for influenza A/H3N2 antigenic prediction based on an ESM-2 protein language model adapted to HA1 sequences.

VirPLM demonstrates superior performance and robustness in both antigenic distance prediction and antigenic variant discrimination. Beyond predictive accuracy, the model offers valuable mechanistic insights by pinpointing high-importance sites enriched in established antigenic regions, such as epitopes A and B. Crucially, VirPLM-prioritized strains achieve higher estimated coverage rates than the corresponding historical WHO-recommended strains in most seasons, highlighting its potential to support candidate vaccine strain prioritization.

While the current model relies on sequence-level representations, its ESM-2 backbone naturally aligns with ESMFold’s structure-prediction capabilities. This shared foundation opens a direct route for multimodal expansion. In future work, integrating structure-informed embeddings into the VirPLM framework promises to further improve the accuracy and interpretability of H3N2 antigenic predictions.

## Supplementary Material

btag692_Supplementary_Data

## Data Availability

VirPLM is freely available through GitHub at https://github.com/xingyili/VirPLM. The exact version of the source code used in this study has been archived in Zenodo (DOI: 10.5281/zenodo.21650323). HA1 sequences of H3N2 influenza viruses can be retrieved from Smith *et al.* (2004) and GISAID (Shu and McCauley 2017) at https://gisaid.org/. Corresponding HI data of H3N2 can be obtained at https://www.crick.ac.uk/research/platforms-and-facilities/worldwide-influenza-centre/annual-and-interim-reports. Additional HI data of H3N2 can be obtained from the study by Smith *et al.* (2004). The HA1 structure of H3N2 (A/Perth/16/2009; PDB ID: 4KVN) is available from the Protein Data Bank (PDB) https://www.rcsb.org.
