# Supplementary material for: VirPLM: antigenic prediction of influenza A/H3N2 viruses with a fine-tuned protein language model
Source: Bioinformatics. 2026 Sep 18;42(10):btag692. doi: 10.1093/bioinformatics/btag692 (PMC13637577; doi:10.1093/bioinformatics/btag692)
Supplement: Supplementary file 1 [file btag692_supplementary_data.pdf]

SUPPLEMENTARY MATERIAL

VirPLM: antigenic prediction of influenza A/H3N2 viruses with a fine-tuned protein language model

1. Supplementary Figures

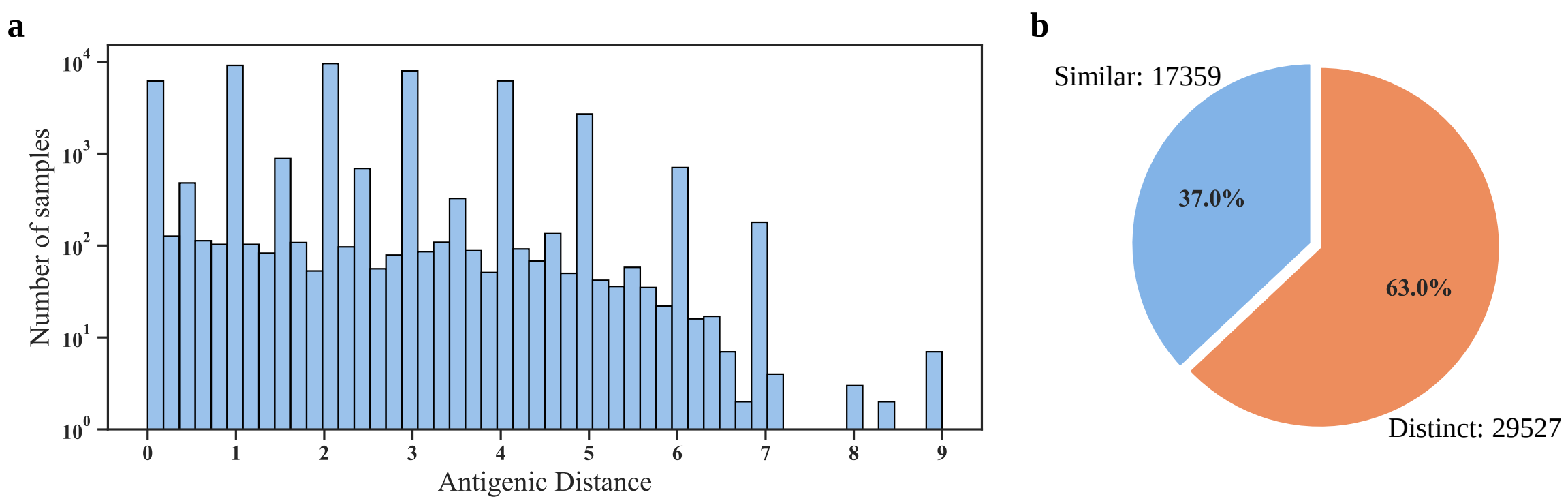

Fig. S1. Overview of the HI data using NAD. (a) Antigenic distance distribution of the HI dataset. (b) Proportion of antigenically similar (distance < 2.0) and distinct (distance ≥ 2.0) antigen-antiserum pairs.

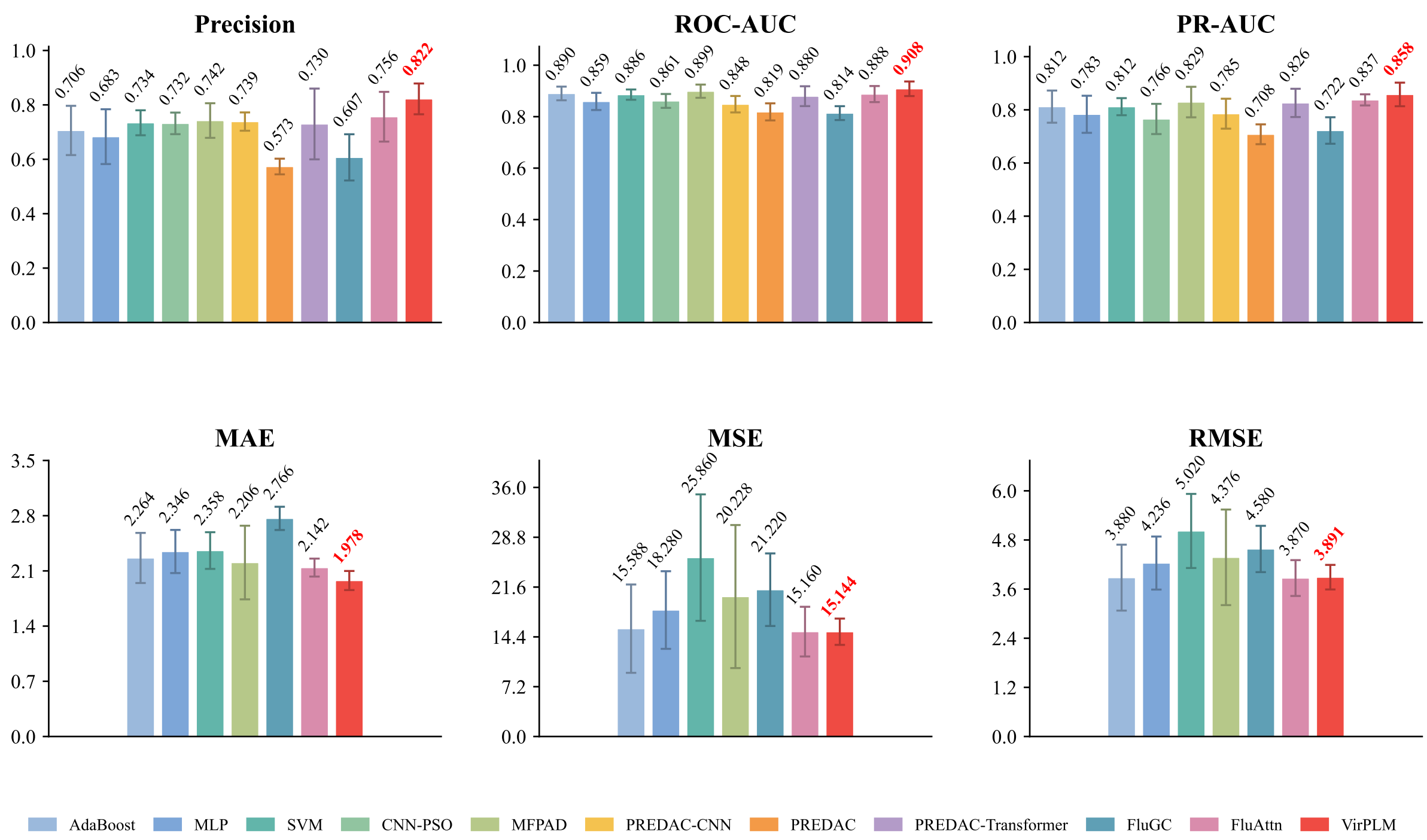

Fig. S2. Five-fold cross-validation performance using AHD. The upper panels show the classification performance of VirPLM and the benchmarked methods. Virus pairs with an AHD of at least 4 are classified as antigenic variants, whereas those with an AHD below 4 are classified as antigenically similar. The lower panels show regression performance. CNN-PSO, PREDAC, PREDAC-CNN, and PREDAC-Transformer are antigenic variant classification models and are therefore not included in the regression comparison.

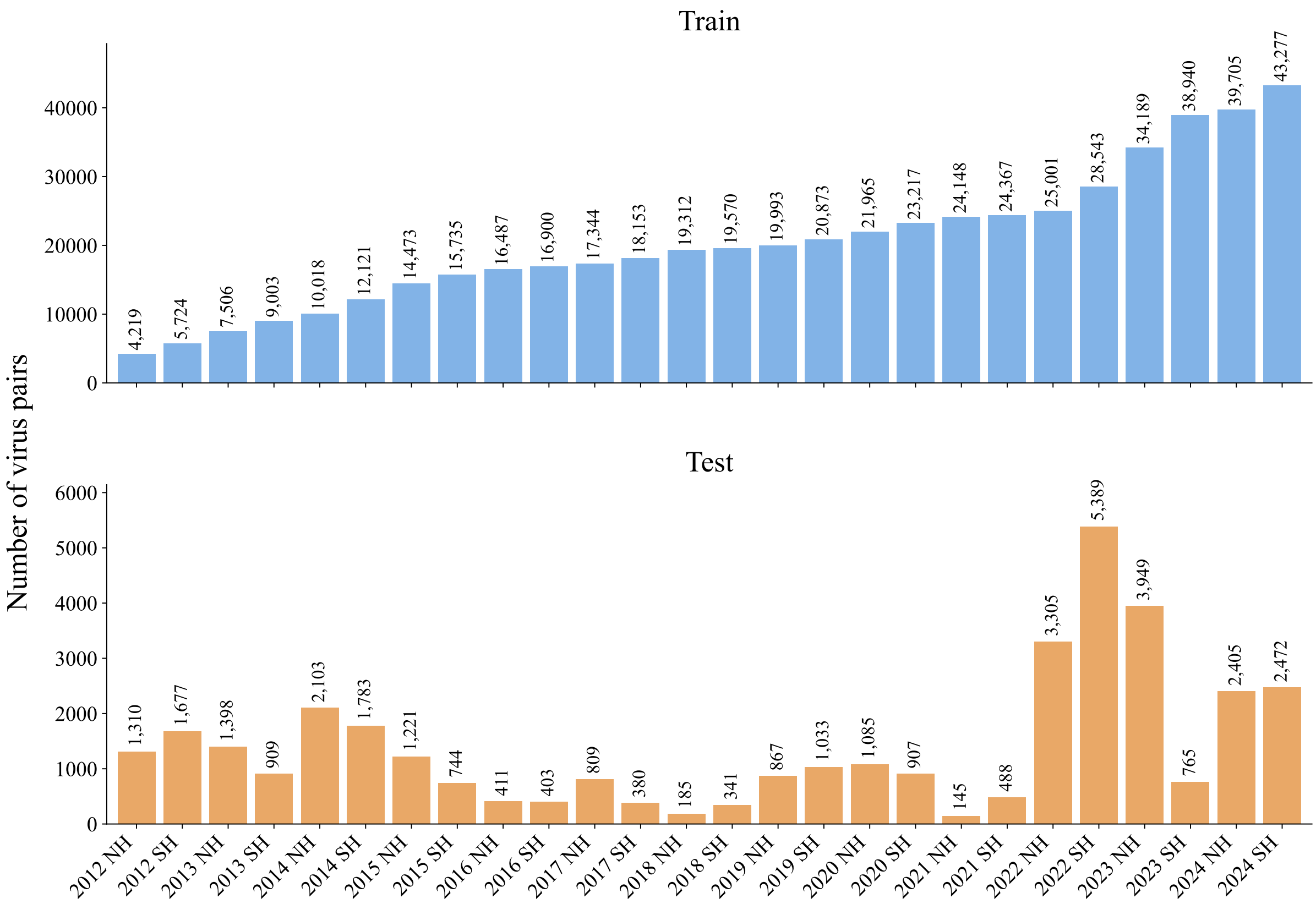

Fig. S3. Numbers of training and test virus pairs using NAD for the retrospective time-split evaluation. The upper and lower panels show the numbers of training and test pairs, respectively, for the 26 seasons from 2012NH to 2024SH. The values above the bars indicate the exact number of virus pairs in each season. NH and SH denote the northern and southern hemispheres, respectively.

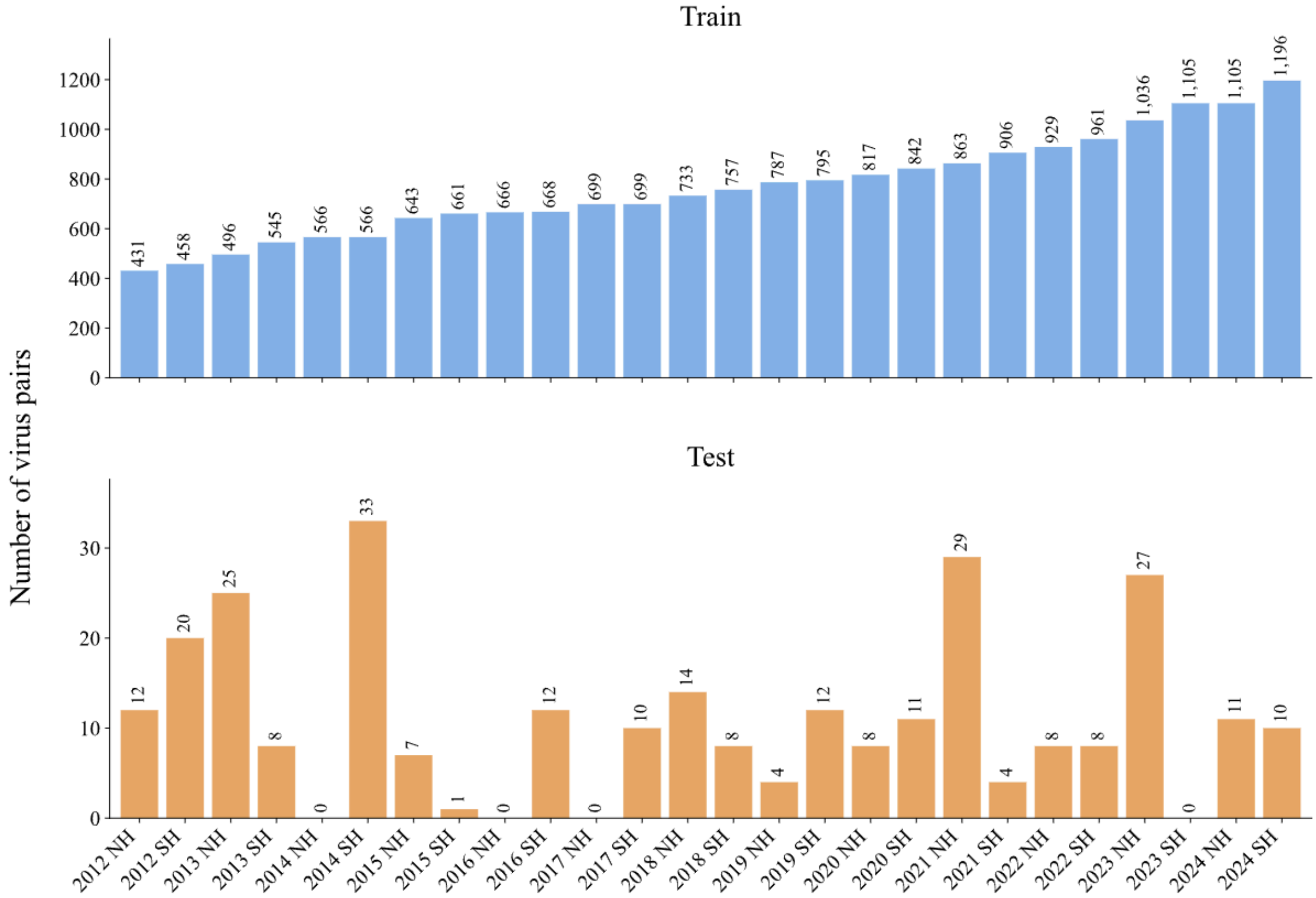

Fig. S4. Numbers of training and test virus pairs using AHD for the retrospective time-split evaluation. The upper and lower panels show the numbers of training and test pairs, respectively, for the 26 seasons from 2012NH to 2024SH. The values above the bars indicate the exact number of virus pairs in each season.

2. Supplementary Tables

Table S1. Vaccine strains prioritized by VirPLM and WHO-recommended strains across 26 influenza seasons.

Season	VirPLM-prioritized strain	WHO-recommended strain
2012NH	A/Maryland/02/2012	A/Victoria/361/2011
2012SH	A/Pennsylvania/14/2010	A/Perth/16/2009
2013NH	A/Kilifi/033/2012	A/Texas/50/2012
2013SH	A/Alabama/04/2011	A/Victoria/361/2011
2014NH	A/Bayern/1/2014	A/Texas/50/2012
2014SH	A/Kilifi/033/2012	A/Texas/50/2012
2015NH	A/California/4393/2015	A/Switzerland/9715293/2013
2015SH	A/Jordan/30677/2013	A/Switzerland/9715293/2013
2016NH	A/SouthAustralia/9/2015	A/Hong Kong/4801/2014
2016SH	A/Paris/2305/2014	A/Hong Kong/4801/2014
2017NH	A/Texas/192/2016	A/Hong Kong/4801/2014
2017SH	A/Brisbane/1001/2016	A/Hong Kong/4801/2014
2018NH	A/Sydney/205/2017	A/Singapore/INFIMH-16-0019/2016
2018SH	A/Ontario/RV1370/2017	A/Singapore/INFIMH-16-0019/2016
2019NH	A/SantaCatarina/487/2018	A/Kansas/14/2017
2019SH	A/NorthCarolina/32/2017	A/Switzerland/8060/2017
2020NH	A/Kanagawa/ZC1841/2019	A/Hong Kong/2671/2019
2020SH	A/Sydney/182/2018	A/South Australia/34/2019
2021NH	A/Parma/31/2020	A/Cambodia/e0826360/2020
2021SH	A/Darwin/113/2020	A/Hong Kong/2671/2019
2022NH	A/Darwin/6/2021	A/Darwin/9/2021
2022SH	A/Darwin/6/2021	A/Darwin/9/2021
2023NH	A/Santiago/88746/2022	A/Darwin/9/2021
2023SH	A/Lisboa/80/2022	A/Darwin/9/2021
2024NH	A/Singapore/GP1355/2023	A/Thailand/8/2022
2024SH	A/Catalonia/NSVH198263302/2022	A/Thailand/8/2022

3. Supplementary Notes

S1. Implementation details of benchmarked methods

AdaBoost (Shah et al. 2024). This method represents each virus pair by encoding amino acid substitutions between their HA1 sequences using an AAindex2 mutation matrix. The feature vector constructed for each virus pair serves as the input to AdaBoost for antigenic prediction. The hyperparameters are set as follows: `n_estimators = 230`, `learning_rate = 1.392`, `max_depth = 1860`, and `max_features = 0.394`.

MFPAD (Li et al. 2024). MFPAD represents HA1 sequence differences of virus pairs using binary encoding and further incorporates 12 types of sequence-derived information related to antigenicity, including mutation counts, changes in N-linked glycosylation sequons, substitutions at key antigenic sites and within antigenic regions A–E, and physicochemical changes in hydrophobicity, volume, charge, and polarity. The resulting feature representation is used to train XGBoost for antigenic prediction. The hyperparameters are set as follows: `max_depth = 7`, `learning_rate = 0.1`, and `n_estimators = 200`.

PREDAC (Du et al. 2012). PREDAC represents each virus pair by 12 HA1-derived structural and physicochemical difference features, including amino acid differences in the five antigenic epitopes, changes in hydrophobicity, volume, charge, polarity, and accessible surface area, changes in predicted glycosylation sites, and the spatial proximity of mutated sites to the receptor-binding region. These continuous scores are discretized to form a binary feature matrix, which is then used to train a naive Bayes classifier for predicting antigenic relationships. Following the original decision rule, virus pairs with an odds ratio greater than 1 are regarded as antigenically similar.

PREDAC-CNN (Meng et al. 2024). PREDAC-CNN represents paired HA1 sequences as an input matrix using a seven-feature encoding scheme, which combines amino acid identity with six physicochemical properties related to antigenicity, including accessible surface area, charge, hydrophobicity, hydrophobicity index, polarity, and volume. The input matrix is fed into a CNN to extract local representations from neighboring HA1 sites and predict antigenic relationships.

CNN-PSO (Lee et al. 2020). CNN-PSO constructs pairwise sequence-difference features from selected HA sites. Candidate amino acid sites are selected using conservation scores, logistic regression, and mutual information, and sequence differences at these sites are encoded using optimized AAindex2 substitution matrices and AAindex3 contact-potential matrices. A CNN is then used to classify virus pairs as antigenic variants or non-variants, with particle swarm optimization (PSO) applied to select AAindex matrices and optimize the CNN architecture.

FluGC (Liu et al. 2025b). FluGC represents influenza strains as nodes in an antigenic relationship graph and uses graph contrastive learning to obtain strain-level representations. Two augmented graph views are generated through edge removal and node-feature masking, and a shared graph attention network encoder is optimized using a contrastive objective. The learned node embeddings are subsequently processed by a positional encoding-based self-attention module and a multilayer perceptron to predict antigenic distances. In the retrospective time-split evaluation, the antigenic relationship graph is reconstructed independently for each target season using only the virus nodes and antigenic-relation edges visible under the corresponding temporal cutoff.

PREDAC-Transformer (Liu et al. 2025a). PREDAC-Transformer represents paired HA1 sequences by combining seven amino acid identity and physicochemical features with ESM-2 embeddings. The resulting pairwise feature matrix is processed by a Transformer encoder to capture long-range dependencies among HA1 sites, followed by fully connected layers and a softmax classifier for predicting antigenic relationships. The model uses two Transformer layers, with eight attention heads.

FluAttn (Geng et al. 2026). FluAttn represents each HA1 sequence pair using position-

wise binary differences together with amino acid property descriptors derived from AAindex1 physicochemical indices and AAindex2 substitution matrices. A two-stage multi-head static attention mechanism is applied separately to the two AAindex sources: the first stage selects the five highest-weighted properties from each source, and the second stage refines their contribution weights. The weighted AAindex1 and AAindex2 representations are concatenated with the binary difference vector and fed into an MLP with hidden dimensions of 1024, 256, and 64 to predict antigenic distances.

Support vector machine (SVM). This baseline uses the same pairwise HA1 representation as AdaBoost, with amino acid substitutions encoded by the AAindex2 mutation matrix. The resulting feature vectors are used to train an SVM for antigenic prediction. The hyperparameters are set as follows: `C = 1.0`, `kernel = 'rbf'`, and `epsilon = 0.1`.

Multilayer perceptron (MLP). This baseline uses the same pairwise HA1 representation as AdaBoost, with amino acid substitutions encoded by the AAindex2 mutation matrix. The resulting feature vectors are used to train an MLP for antigenic prediction. The hyperparameters are set as follows: `alpha = 0.0001`, and `learning_rate_init = 0.001`.

S2. Temporal data partitioning for retrospective evaluation and vaccine strain prioritization

All date ranges described below include the start date and exclude the end date.

S2.1. Retrospective time-split evaluation of antigenic prediction

The retrospective antigenic prediction evaluation comprises 26 test seasons from 2012NH to 2024SH. A separate VirPLM model is trained and evaluated for each season, and both virus-specific MLM pretraining and supervised fine-tuning are restricted to data collected before the corresponding target-season window.

For a northern hemisphere season in year T, 1 September of year $T-1$ is used as the temporal cutoff. The supervised training set contains antigen–antiserum pairs for which both the antigen virus and the reference virus used to raise the antiserum were collected before 1 September of year $T-1$. The test set contains antigen viruses collected from 1 September of year $T-1$ to 1 February of year T, paired with antisera raised against reference viruses collected before 1 September of year $T-1$.

For a southern hemisphere season in year T, 1 February of year T is used as the temporal cutoff. The supervised training set contains antigen–antiserum pairs for which both the antigen virus and the reference virus used to raise the antiserum were collected before 1 February of year T. The test set contains antigen viruses collected from 1 February to 1 September of year T, paired with antisera raised against reference viruses collected before 1 February of year T.

For each season-specific model, MLM pretraining uses only quality-controlled HA1 sequence records collected before the corresponding cutoff date, whereas supervised fine-tuning uses only the historical antigen–antiserum pairs defined above.

S2.2. Decision-time vaccine strain prioritization

The vaccine strain prioritization analysis also comprises 26 target seasons from 2012NH to 2024SH. 1 February and 1 September are used as fixed cutoff dates for the northern- and southern-hemisphere analyses, respectively. These dates correspond to the months in which the WHO vaccine composition consultations are typically held for the two hemispheres (World Health Organization 2023, 2024).

For a northern hemisphere target season in year T, 1 February of year T is used as the decision-time cutoff. Candidate strains are restricted to viruses collected from 1 February of year $T-1$ to 1 February of year T. They are ranked according to their predicted coverage of surveillance viruses collected from 1 September of year $T-1$ to 1 February of year T, with the frequency-weighted mean NAD used as the secondary ranking criterion when candidate strains have identical coverage rates.

For a southern hemisphere target season in year T, 1 September of year $T-1$ is used as the decision-time cutoff. Candidate strains are restricted to viruses collected from 1 September of year $T-2$ to 1 September of year $T-1$. They are ranked according to their predicted coverage of surveillance viruses collected from 1 February to 1 September of year $T-1$, using the same primary and secondary ranking criteria as in the northern-hemisphere analysis.

S2.3. Postseason evaluation of prioritized vaccine strains

For a northern hemisphere target season in year T, the VirPLM-prioritized strain and the corresponding historical WHO-recommended strain are evaluated against viruses collected from 1 October of year T to 1 April of year $T+1$. For a southern hemisphere target season in year T, the two strains are evaluated against viruses collected from 1 April to 1 October of year T. For each candidate–target virus pair, the experimentally measured NAD is used whenever a valid HI-derived value is available. When no experimental NAD is available, the missing antigenic distance is estimated using a separately trained postseason evaluation model. For each target season, this model is trained using HA1 sequences and HI data associated with viruses collected before the end date of the corresponding postseason evaluation window. This model is introduced only after decision-time candidate ranking has been completed and is used exclusively to estimate missing distances for retrospective performance assessment. No information from the postseason evaluation model is used during candidate selection.

References

Du X, Dong L, Lan Y et al. Mapping of H3N2 influenza antigenic evolution in China reveals a strategy for vaccine strain recommendation. *Nat Commun* 2012;3:709. `https://doi.org/10.1038/ncomms1710`

Geng L, He J, Liu P. FluAttn: antigenicity prediction of influenza A/H3N2 through attention-based feature mining. *Infect Dis Model* 2026;11:428–37. `https://doi.org/10.1016/j.idm.2025.11.005`

Lee EK, Tian H, Nakaya HI. Antigenicity prediction and vaccine recommendation of human

influenza virus A (H3N2) using convolutional neural networks. *Hum Vaccin Immunother* 2020;16:2690–708. https://doi.org/10.1080/21645515.2020.1734397

Li X, Li Y, Shang X et al. A sequence-based machine learning model for predicting antigenic distance for H3N2 influenza virus. *Front Microbiol* 2024;15:1345794. https://doi.org/10.3389/fmicb.2024.1345794

Liu J, Wang J, Wang C et al. Deciphering the antigenic evolution of seasonal influenza A viruses with PREDAC-Transformer: from antigenic clustering to key site identification. *bioRxiv*, 2025a, preprint: not peer reviewed. https://doi.org/10.1101/2025.10.29.684974

Liu Q, Xia Y, Guo Y et al. Graph contrastive learning with positional embeddings for predicting influenza antigenicity. *Inf Sci* 2025b;733:122955. https://doi.org/10.1016/j.ins.2025.122955

Meng J, Liu J, Song W et al. PREDAC-CNN: predicting antigenic clusters of seasonal influenza A viruses with convolutional neural network. *Brief Bioinform* 2024;25:bbae033. https://doi.org/10.1093/bib/bbae033

Shah SAW, Palomar DP, Barr I et al. Seasonal antigenic prediction of influenza A H3N2 using machine learning. *Nat Commun* 2024;15:3833.

World Health Organization. Recommended composition of influenza virus vaccines for use in the 2024 Southern Hemisphere Influenza Season. Meeting report, World Health Organization, 2023.

World Health Organization. Recommended composition of influenza virus vaccines for use in the 2024–2025 northern hemisphere influenza season. *Wkly Epidemiol Rec* 2024;99(12):135–146.
